# Factors associated with birth preparedness and complication readiness in Southern Ethiopia: a community based cross-sectional study

**DOI:** 10.1186/s12884-017-1582-3

**Published:** 2017-12-08

**Authors:** Eshetu Andarge, Aderajew Nigussie, Mekitie Wondafrash

**Affiliations:** 1 Department of Reproductive Health and Nutrition, School of Public Health, College of Health Sciences and Medicine, Wolaita Sodo University, P.O. Box: 138, Wolaita, Ethiopia; 2 Department of Population and Family Health, Faculty of Public Health, Jimma University, P.O. Box: 378, Jimma, Ethiopia

**Keywords:** Birth preparedness, Complication readiness, Pregnant women, South Ethiopia

## Abstract

**Background:**

Birth preparedness and complication readiness (BP/CR) is a strategy to promote use of skilled maternal and neonatal care so that they can get timely skilled care, particularly during child birth. There is minimal evidence on the factors associated with BP/CR among pregnant women in Ethiopia. Hence, this study aimed to assess the factors influencing BP/CR among pregnant women in Southern Ethiopia for the purpose of improving utilization of skilled attendant at birth.

**Methods:**

A community based cross-sectional study was conducted among 707 pregnant women in Southern Ethiopia in March 2015. Both quantitative and qualitative methods of data collection were used. For the quantitative study, the study subjects were included in the study by employing multi-stage sampling. Data was entered into Epidata version 3.1 and analyzed using IBM SPSS statistics 20. Level of statistical significance was declared at a p- value of <0.05. For the qualitative study, six FGDs were conducted and analyzed based on the thematic areas.

**Result:**

The prevalence of BP /CR in Arba Minch Zuria Woreda was found to be 30%. The odds of being prepared for birth and its complications was higher among women from high economic class (AOR = 2.29, 95% CI = 1.16, 4.54), with frequency of antenatal care(ANC) > = 4 (AOR = 4.52, 95% CI = 2.26, 9.02), who received advice on BP &CR (AOR = 1.84, 95% CI = 1.13, 3.01),and who were knowledgeable on labor and delivery(LAD) danger signs (AOR = 1.85, 95% CI = 1.01, 3.44). However, it was lower among women with parity of 2 - 4(AOR = .0.51, 95% CI = 0.31, 0.84) and >4 (AOR = 0.51, 95% CI = 0.31, 0.84) than primiparous women. It was also lower among women from food insecure households (AOR = 0.26, 95% CI = 0.16, 0.42) than their counterparts. Lack of awareness on BP/CR, privacy and respect in health institutions were mentioned by the FGD discussants as barriers to women’s preparation for birth.

**Conclusions:**

The study showed that BP/CR is inadequate among pregnant women in the study area. Improving socio-economic and food security status of women, strengthening community-based education on complete attendance of ANC, and conforming to professional ethical standards were recommended.

**Electronic supplementary material:**

The online version of this article (10.1186/s12884-017-1582-3) contains supplementary material, which is available to authorized users.

## Background

Majority of the global maternal deaths (99%) occurred in developing regions in 2015, about two-third (66%) of which occurred in sub-Saharan Africa alone. Ethiopia is one of the ten countries that comprised 59% of the global maternal deaths with 11,000 deaths. However, the country accomplished MDG 5 “making progress” with Maternal Mortality Ratio (MMR) of 353 per 100,000 live births with reduction by 71.8% from the 1990s estimate slightly short of the target of 75% [1]. The Sustainable Development Goals (SDGs) with a vision of ending all preventable maternal mortality by 2030 further calls for an acceleration of current progress with the target of achieving a global MMR of 70 per 100, 000 live births, or less. In line with the global aspirations, Ethiopia has currently set a five years health sector transformation plan (HSTP) to reduce MMR to 199 per 100,000 live births by 2020 [1, 2]. In Ethiopia, the causes of maternal deaths are similar to many developing countries as they stem from the three delays; delay in seeking care, delay in reaching appropriate care and delay in receiving care [3].

Many of the complications that result in maternal and perinatal deaths are unpredictable, and their onset can be both sudden and severe [4–6]. Hence, timely access to skilled care is important to women and newborns prior to, during and after childbirth. Too often, however, their access to care is impeded by delays [7]. Delay in responding to the onset of labor and the complications have been shown to be one of the major barriers to reducing mortality and morbidity surrounding child birth [4, 6]. As far as delay in receiving skilled obstetric care is concerned, lack of timely planning for use of a skilled birth attendant for normal births and inadequate preparation for prompt action in the event of obstetric complications are well documented factors. Birth preparedness and complication readiness (BP/CR) is a strategy to promote the timely use of such a care [5]. Making a birth plan before and during pregnancy has shown to facilitate a feeling of self-control and autonomy for pregnant women which in-turn have shown a positive impact on pregnancy and birth outcomes [4].

Although measurements vary from place to place, in many developing countries including Ethiopia, BP/CR is not a common practice. About 49.4, 24.7, 86.2 and 53.9% of women were prepared for birth and its complications in West Bengal India, Northern Nigeria, Mpwapwa district of Tanzania and Mbarara District of Southwest Uganda respectively [8–11]. In Northern Ethiopia only 22%, in Bale and Arsi Zones, Central Ethiopia, 29.9 and 16.5% respectively and 17% of women in Southern Ethiopia were prepared for birth and its complications [12–15].

According to the nationwide road map for accelerating the reduction of maternal and newborn morbidity and mortality, one of the main strategies for the reduction of maternal mortality in Ethiopia is increasing community awareness of BP/CR [16]. As a result of this, increased efforts on ensuring implementation of the BP/CR have been made by introducing focused antenatal care (FANC) which emphasizes counseling of women on the elements of this strategy. High impact interventions like free maternal and ambulance services have been in place following the government’s effort to achieve MDG 5 [17]. The government has also extended its commitment in intensifying Reproductive, Maternal, Neonatal, Child, Adolescent and Youth Health (RMNCAH) interventions to end preventable maternal and child deaths by 2030 in line with the global aspirations of SDGs. RMNCAH and nutrition will continue to be top priority in the country’s health sector transformation plan [2].

However, according to Ethiopian Demographic and Health survey (EDHS) 2011, the three successive survey reports showed no evidence to suggest that the estimated maternal mortality ratio decreased in Ethiopia between 2000 and 2011 [18]. Studies have shown that BP/CR is a strong predictor of institutional/ health facility delivery [19, 20]. Skilled birth attendance is the most important proven intervention in reducing maternal mortality [21, 22] and one of the MDG indicators to track national effort towards safe motherhood [23]. There is a wide gap in proportion of pregnant women’s ANC attendance (40%) and institutional delivery in Ethiopia. In the last fifteen years, skilled assistance at delivery increased only by 9% (from 6 to 15%). Southern Nations and Nationalities Region (SNNPR) was one of the regions with the lowest proportion of births assisted by a skilled provider (12%) [24]. In our study area as well health facility delivery was the least in Gammo Goffa Zone (19%) [25]. This negligible decline in MMR and increase in institutional delivery in the successive EDHS reports could be related to low use or non-use of BP/CR strategies recommended by the World health organization (WHO).

As a factor of adequacy or the dietary intake in pregnancy, the nutritional status of women, is an important contributor to good pregnancy outcome. Household food security is a determinant of adequate dietary intake and it is a major and ever worsening problem in Ethiopia [26, 27]. In addition to its direct effect on pregnancy outcomes, different literatures showed that household food insecurity has also shown statistically significant association with health care utilization like antenatal care and accessing skilled delivery care [28–30]. Hence, these negative effects may be understood through the concept of competing demands which could impose precedence of food over health care. People may be averted to place limited financial resources or time in food or housing than they do for health care as food is the most basic need. This was particularly evidenced among homeless populations and persons with HIV infection [29]. Since pregnant women are also vulnerable groups of the community, they may also be obliged to choose spending limited financial resources or time in food or housing than striving to get the necessary health care whenever they are in food insecure conditions. One of such effects would be operating through preparing for birth and its complications.

However, to the best of the researcher’s knowledge, there is no evidence which supports the association between BP/CR and household food security status in Ethiopia in general and in the study area in particular and this study considers food insecurity as a single factor which might affect preparation for birth and its complications. Hence, the study will contribute to the knowledge base whether nutritional factors like food insecurity and social factors like exposure to mass media have association with birth preparedness and complication readiness or not. This study also sets women’s socio-economic status using a wealth index based on their assets unlike previous studies [13–15] which used average monthly income after converting to Ethiopian Birr which may not give a valid measure among rural women.

Even though a previous study was conducted in Southern Ethiopia [15] in 2007, it was immediately after the implementation of focused antenatal care in the country. There was no current estimate of BP/CR after implementation of the high impact interventions like free maternity and ambulance services [17] in the local context. Hence, there was a need to conduct a recent study in order to estimate BP/CR and factors associated with it to identify gaps for policy and program improvement in line with the SDGs. Moreover, few of the studies conducted in the country employed only quantitative method of data collection [12, 13, 15]. Qualitative data with regard to exploring women’s knowledge on BP/CR and factors affecting their preparation are limited [14]. Thus, to fill these gaps, the present study was conducted in Southern Ethiopia.

## Methods

### Study setting and design

The study was conducted in March 2015 in Arba Minch Zuria Woreda, one of the woredas in Gammo Goffa Zone, South Ethiopia. The woreda consists of 29 rural kebeles (smallest administrative unit in the Ethiopian government administration). According to the Woreda‘s health office report, the total population of the woreda projected for the year 2007 E.C (2014/2015 G.C) was 205,204. Pregnant women in the woreda were estimated to be about 7101. The Woreda has 6 health centers and 37 health posts. The Woreda‘s main town is Arba Minch which is the capital town of Gammo Goffa zone. It is located 505kms far to the South of Addis Ababa, capital city of Ethiopia. Farming is the predominant occupation of residents in the woreda. Low land areas of the woreda are the major producers of banana supplying the markets in big cities in Ethiopia. Two of the Rift Valley lakes (Abaya and Chamo) are also situated at the Eastern border of the Woreda. It is also one of the tourist destinations in Ethiopia having Nech Sar National Park on the plain at the back of a mountain bridging the two lakes, namely `Ye Egzier Dildiy’ meaning the bridge of God. The forty springs, a spring in the forest at the Eastern border of Arba Minch town is also one of the attraction areas in the woreda [25].

A community- based cross-sectional study was conducted among pregnant women with a self-reported pregnancy of 3 months and above who were randomly selected from 9 kebeles in the woreda. During data collection, women who were seriously sick or unable to give information and who lived in the study area for less than 6 months were excluded.

### Sample size and sampling procedure

For the quantitative survey, the sample size was calculated using single population proportion formula assuming 17% proportion of birth preparedness among pregnant women in the zone [15], 95% confidence, and a margin of error of 4%. The sample size calculated with this consideration was 339**.** After applying finite population correction formula and 10% non-response rate and design effect of 2, the final sample size was 713.For the qualitative data, a total of six focus group discussions (FGDs) were formed in groups of husbands of pregnant women, traditional birth attendants (TBAs) and health professionals. Each of the groups consisted 8 to 12 members.

There are a total of 29 rural kebeles in Arba Minch Zuria Woreda [25]. Pregnant women in the woreda were estimated to be about 7101 which were 3.46% of the total population (3.46% is a conversion factor for estimated number of pregnancies for SNNPR in Ethiopia). Hence, the estimated number of pregnant women for each kebele was obtained by multiplying the total population of the kebele by 3.46%. Multi-stage sampling was used to select the study subjects. In the first stage, nine kebeles were selected from the 29 kebeles randomly. The sample was proportionally allocated to each kebele with consideration of the estimated number of pregnant women per each kebele. Then using family folder (a folder containing different health and health related information of a family) in the health posts of each kebele, pregnant women were listed in each kebele. From the list, the required number of pregnant women in each kebele was selected randomly by using IBM SPSS statistics 20. For the qualitative data, non-probability (purposive) sampling technique was used to obtain homogenous groups for each category (husbands, TBAs, and health professionals).

### Data collection method, tools and procedure

A total of 7 public health nurses and 2 health officers were recruited for the quantitative data collection and supervision of the collection process, respectively. Both data collectors and supervisors were given a day long intensive training on the data collection methods. A pretested, structured, interviewer administered questionnaire was used. The questionnaire was partly adapted from the survey tools developed by JHPIEGO Maternal and Neonatal Health program [5] and household food insecurity status of women was assessed using the standard Household Food Insecurity Access Scale (HFIAS) questionnaire by Food And Nutrition Technical Assistance (FANTA III) [31]. During the data collection period, the data collectors and supervisors were guided by health development army (HDA) leaders in each kebele so that they can easily access the houses of each sampled pregnant woman. The data collectors were given the list of the pregnant women to be interviewed in their respective kebeles in advance.

For the qualitative data, unstructured open ended and non-directive FGD guide was designed in order to triangulate responses obtained from the quantitative survey. The principal investigator moderated the discussion of health professionals, while the TBAs and the husband‘s group were moderated by an experienced health officer working in the woreda in the presence of note takers and technical assistants. A day long training and practical exercise was carried out before the commencement of the data collection. Group discussions with their respective discussants were conducted in a quiet kebele and health center halls. Each discussion was tape recorded not to miss all issues discussed.

### Data analysis

Data was entered in to Epidata version (3.1) and analyzed using IBM SPSS statistics 20. Descriptive statistics using frequencies, percentages, mean, and standard deviations was used to describe findings. Bivariate analysis using logistic regression was done and all explanatory variables which have association with the outcome variable at *p*- *value* of less than 0.25 were selected as candidates for multivariable analysis. Multi-collinearity between the candidate variables was checked. Then multivariable analysis using back ward stepwise selection method was done to control for possible confounding variable and to determine presence of statistically significant association between explanatory variables and the outcome variable. Level of statistical significance was declared at a *p*-value of <0.05 and OR with 95% CI was used to measure the degree of association between independent variables and the outcome variable. Model fitness was checked using Hosmer and Lemeshow goodness of fitness test. Hence, a total of 13 variables with a *P*-value less than 0.25 in the bivariate logistic regression analysis (age of women, educational status, marital status, occupation, ever listened to the radio, history of still birth, frequency of ANC, advice during ANC, place of delivery for the last birth, knowledge on key pregnancy, labor and delivery and post- partum danger signs, household food security status) were entered in to a multivariable logistic regression model. Factor analysis was conducted to set wealth /economic status of pregnant women. A total 13 items on household assets were analyzed using principal component analysis method after checking the fulfillment of assumptions using Kaiser-Meyer-Olkin measure of sampling adequacy and Bartlett’s Test of Sphericity.

The qualitative data was analyzed manually by the principal investigator following the steps of frame work analysis [32].However, all authors read the material and reflected on each of the transcripts, codes, subcategories, categories and themes until agreement was reached. Prior to analyzing the data, all FGDs were transcribed in *Gamotho* and then translated into English by the first author and a research assistant (moderator of the husband’s and TBAs group). Preliminary coding of transcripts was done and consistent codes were condensed in to categories and subsequently in to themes. Finally, the categories and themes emerged from the data were identified and were presented together with powerful quotes in narrative ways by triangulating with quantitative data.

### Data quality management

Various activities were performed to assure the quality of data. The English version questionnaire was translated to the local language (Gammotho) by a person having knowledge of both of the languages. Then another individual who had very good knowledge of both English and Gammotho language translated the *Gammotho* version back to English to check for its original meaning. The questionnaire was pre-tested on 36 respondents (5% of sample size) in *Chano Dorga* kebele that had similar characteristics with the study population other than the sampled cluster in the study area. Findings were discussed among data collectors, supervisors and the investigator in order to ensure better understanding to the data collection process. Based on the pretest, questions were revised, edited, and those found to be unclear or confusing were modified. Finally, a structured *Gammugna* version questionnaire was used for data collection. The principal investigator and supervisors made supervision on the data collection process. Every day, 10% of the completed questionnaires were reviewed and checked for completeness and consistency by the supervisors and principal investigator and the necessary feedback offered to data collectors in the next morning before the data collection begins. Each woman was interviewed in a separate private place to avoid social desirability bias. For the qualitative data, the kebele administrators, health administrators, and the supervisors, with principal investigator identified eligible discussants which were supposed to be informative from each group.

### Definitions and measurement

Kebele: refers to the smallest administrative unit in the Ethiopian context.

Woreda: refers to an administrative unit above kebele.

Family folder: It is a household centered service delivery tool or registration book that contains information on household characteristics and other health and health related information of members of the household which is available at the health posts.

A woman was considered as prepared for birth and its complication if she reported that she saved money (planned to),identified health facility(planned to) for delivery (health center/hospital),, identified skilled provider at birth(planned to) and identified a means of transport to place of childbirth or for the time of obstetric emergencies(planned to) ahead of childbirth. Those mothers who reported that they have followed (planned to) at least three of the four BP/CR components were considered as ―well prepared for birth and its complication. The remaining women were considered as ―not well prepared for birth and its complications.

A woman was considered knowledgeable on key danger signs of pregnancy, if she can mention at least two of the three key danger signs for pregnancy (vaginal bleeding, swollen hands/face and blurred vision) spontaneously [14].

A woman was considered knowledgeable on key danger signs of labor/childbirth, if she can mention at least three of the key four danger signs for labor/childbirth (severe vaginal bleeding, prolonged labor (>12 h), convulsion and retained placenta) spontaneously [14].

A woman was considered knowledgeable on key danger signs of postpartum, if she can mention at least two of the three key danger signs for postpartum (severe vaginal bleeding, foul-smelling vaginal discharge and high fever) spontaneously [14].

A mildly food insecure household worries about not having enough food sometimes or often, and/or is unable to eat preferred foods, and/or eats a more monotonous diet than desired and/or some foods considered undesirable, but only rarely [31].

A moderately food insecure household: eats a monotonous diet or undesirable foods sometimes or often, and/or has started to cut back on quantity by reducing the size of meals or number of meals, rarely or sometimes [31].

A severely food insecure household: Any household that experiences one of these three conditions(running out of food, going to bed hungry, or going a whole day and night without eating) even once in the last 4 weeks (30 days) [31] .

A household was considered as food insecure when it has mild, moderate or severe food insecure conditions whereas a food secure household experiences none of the food insecurity conditions or just experiences worry, but rarely [31].

### Ethical approval and consent to participate

Ethical clearance was obtained from Jimma University Ethical Review Committee (Ref.No. PFHD/055/677/07). A formal letter of permission to conduct the study was obtained from Gammo Goffa zone health desk and subsequently from Arba Minch Zuria Woreda Health office (Ref.No.  + 5/2799/2). Verbal consent was obtained from the study subjects and they were informed that the data will be kept confidential.

## Result

In this study a total of 707 pregnant women with a self-reported pregnancy of 3 months and above were interviewed from the randomly selected kebeles in Arba Minch Zuria Woreda comprising a response rate of 99.2%.

### Prevalence of birth preparedness and complication readiness

Majority of the respondents 626 (88.5%) mentioned saving money as a means of preparing for birth and its complications. However, less than a third of them mentioned identifying skilled provider 193(27.3%) as way of preparing for birth and its complications. Only few mentioned identifying blood donor as a way of preparation for birth and its complications 41(5.8%). Regarding the arrangements made 502(71.0%) of the pregnant women in the study made some arrangements for birth and its complications (Table 1).

**Table 1:** Awareness and practice of pregnant women on preparation for birth and its complications in Arba Minch Zuria Woreda, South Ethiopia, March 2015

Variables	Category	Frequency (%)
Know elements of Birth preparation (N = 707)	Saving money	626(88.5)
	Identifying mode of transportation	285(40.3)
	Identifying skilled provider	193(27.3)
	Identifying place of delivery	313(44.3)
	Identifying blood donor	41(5.8)
	Others	73(10.3)
Made arrangements for birth and its complications(N = 707)	Yes	502(71.0)
	No	205(29.0)
BP/CR (n = 707)	Well-prepared	212(30.0)
	Not well-prepared	495(70.0)

*BP/CR* Birth preparedness and complication readiness

*Others* Porridge goods, slaughtering animals, new blade, etc.

In the FGDs, majority of the discussants reported that they believe in the importance of preparation for birth for a healthy pregnant woman since they obviously observed that a labor is not predictable, mentioning its occurrence in the midnight, when there is no one with the woman, with severe bleeding, without having money to go to health facilities or the nearby road for transport access.

A 43 years old man, one of the discussants in the men FGD said *“I remember the moment when a torrential rain with thunderstorm began in the midnight and all of a sudden my wife began to suffer from a labor pain, I was highly keyed up by the situation. I don’t have a helper in the home except two of my children who were very young and the labor lasted the whole night and she passed the night shouting. On the coming day we waited the whole day expecting for a normal birth. We had no money in hand as it was not a harvesting season for all of us and also we had no access for transportation here in the high lands. After she has been exhausted, we took her to the hospital and we were told that she needs an operation because her uterus was torn by that time. She lost her uterus and we were left with only two children.Hence, we advise the importance of preparation for birth for the whole community as it was painful to cope with such catastrophic incidents.”*

**Fig. 1:**
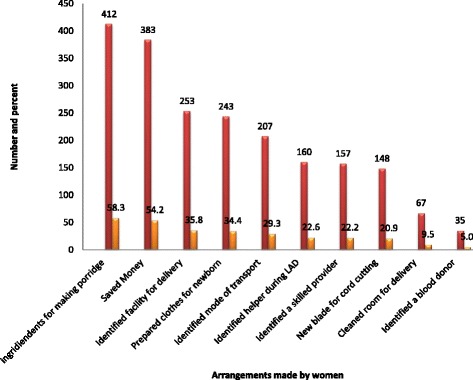
Arrangements made by pregnant women for birth and its complications in Arba Minch Zuria Woreda, South Ethiopia, March 2015

The most common preparation was made for ingredients of porridge (the commonest dish served for a postnatal mother in South Ethiopia) where 412(82.1%) women prepared the ingredients like cereals, cooking oil, milk and butter. Among the important components considered for birth preparedness and complication readiness, the most common preparation was made for saving money 383(76.3%). This accounts for 54.2% of total number of women participated in the study (Fig. 1). Over all, only 212(30%) of the pregnant women were well prepared for birth and its complications and 495(70.00%) of them were not well prepared for birth and its complications (Table 1).

Majority of the FGD discussants agreed on preparations for porridge, and saving money as their practice of birth preparedness. From the husband’s FGD, a 39 years old man said *“we have four children…so far we think that we were prepared for birth when we fulfill materials for porridge, new blade for cord cutting, butter for feeding the baby, and saving money, but till now she didn’t face any problem. We were using the money for buying a slaughtering animal, and cloth for the baby.”*

Most TBAs in the TBAs discussion raised the issue of lack of privacy and respect in health facilities which they mentioned as the main reason women come to get service from them, and do not prepare for giving birth in the health facilities*.* A 42 years old lady, one of the discussant in the TBAs FGD said*, “Women come to us for birth, not because they knew that we are experienced than the health professionals rather they know that we treat them friendly, keeping their privacy. Those women who give birth in our hands are ashamed of visiting health institutions where males, students, and a group of females watch them naked. This is out of our culture; they come to us even being late after passing several hours waiting for a normal birth……..but finally when they become between life and death,the whole community starts to give attention. In some households their husbands do not allow them to visit health institutions for the reason I mentioned earlier.”*

### General characteristics of the study participants

Four hundred seventy three (66.9%) of the respondents were in the age group of 20–34 years, with a mean age of 27.2 ± (5.5) years. Regarding their marital status, 676 (95.6%) were married and the remaining few 31(4.4%) were not in a marital union. Around half of the study participants were house wives 338(47.8%) followed by farmers 249 (35.2%). Two hundred fifty four (35.9%) of the respondents were illiterate and more than one third 287(40.6%) of them attended primary school level education. With consideration of women‘s wealth status in quartiles 230(32.6%) of women had high wealth status. More than half 370 (52.3%) of the study participants ever listened to the radio (Table 2).

**Table 2:** Socio-demographic characteristics of pregnant women in Arba Minch Zuria Woreda, South Ethiopia, March 2015

Variable	Category	Frequency (%)
Age of women(N = 707)	less than 20	89(12.6)
	20-34	473(66.9)
	35 and above	145(20.5)
	Mean ± SD	27.2 ± 5.5
Religion(N = 707)	Protestant	434(61.4)
	Orthodox	262(37.1)
	Others^a^	11(1.6)
Marital status(N = 707)	In marital union	676(95.6)
	Not in marital union^d^	31(4.4)
Living with a partner(N = 707)	Yes	661(93.5)
	No	46(6.5)
Ethnicity(N = 707)	Gammo	572(80.9)
	Wollaita	97(13.7)
	Zeise	17(2.4)
	Others^b^	21(3.0)
Occupation(N = 707)	House wife	338(47.8)
	Farmer	249(35.2)
	Others^c^	120(17.0)
Educational status(N = 707)	Illiterate	254(36.0)
	Read and write	71(10.1)
	Primary education	287(40.7)
	Secondary education and above	95(13.4)
Wealth status(N = 705)	Low	203(28.8)
	Low-medium	149(21.1)
	Medium-high	123(17.4)
	High	230(32.6)
Ever listen to the radio(N = 707)	Yes	370(52.3)
	No	337(47.7)
Frequency of listening to the radio(N = 370)	Almost every day	186(50.3)
	Once in a week	158(42.7)
	Less than once in a week	26(7.0)

^a^ Muslims, no religion

^b^ Amhara,Guragie, Oromo

^c^ government employees,students, daily labors, merchants

^d^ single, divorced, separated, widowed

About a third of the women were pregnant before the age of 18 years and 451(63.80%) were above 18 years of age when they were first pregnant with mean age at first pregnancy being 20.2 ± (2.9). Majority of the women 588(83.20%) were pregnant twice and above. Regarding parity of the women, more than half 332(56.90%) of the women with previous pregnancies had parity of two to four. Among women with previous pregnancies 114(19.40%) and 70(11.91%) had ever had abortion and still birth respectively. More than half 338(57.5%) of the pregnant women with previous pregnancies gave their previous birth at home. Only 86(14.6%) of women were assisted by midwives, nurses, health officers and physicians during birth. Hence, the skilled birth attendance was 14.6%. Majority of the respondents 616(87.2%) ever heard about ANC service and 91(12.8%) never heard about ANC service. Among women who heard about ANC service, the majority 502(81.5%) have received the service for this current pregnancy. Only70 (13.9%) of the women attended ANC four and more times. About four hundred sixty one (91.8%) of ANC attendants have received advice during ANC (Table 3).

**Table 3:** Obstetric and antenatal experience of pregnant women living in Arba Minch Zuria Woreda, South Ethiopia, March 2015

Variable	Category	Frequency (%)
Age at first pregnancy(N = 707)	less than 18	194(27.4)
	Greater than or equal to 18	451(63.8)
	Do not know	62(8.8)
	Mean ± SD	20.2 ± 2.9
Gravidity (N = 707)	One	119(16.8)
	2 and above	588(83.2)
Parity(n = 583)	One	203(34.8)
	Two to four	332(56.9)
	Five and more	48(8.2)
Ever had abortion or miscarriage(n = 588)	Yes	114(19.4)
	No	474(80.6)
Ever had still birth(n = 583)	Yes	70(11.9)
	No	513(88.1)
Place of last birth(n = 588)	At home	338(57.5)
	At health institution	250(42.5)
Attendant of birth(n = 588)	Relative/caretaker/TBA	338(57.5)
	Health extension worker	164(27.9)
	Midwife/nurse/health officer/physician	86(14.6)
Ever heard ANC service(N = 707)	Yes	616(87.1)
	No	91(12.9)
ANC visit(n = 616)	Yes	502(81.5)
	No	114(18.5)
Frequency of ANC(n = 502)	Less than four visits	432(86.1)
	Greater than or equal to four visits	70(13.9)
Received advice during ANC(n = 502)	Yes	461(91.8)
	No	41(8.2)

### Knowledge of pregnant women on key danger signs during pregnancy, LAD, and the post-partum period

About 461(65.2%) reported spontaneously that they heard about serious problems/danger signs during pregnancy, almost a similar proportion of women heard about LAD danger signs, however a relatively lower proportion of women 401(56.7%) heard about postpartum danger signs. Among women who heard about danger signs, vaginal bleeding was the most commonly mentioned danger sign during pregnancy 372(80.7%), labor and delivery 393(85.1%) and the post-partum period 345(86.1%). Blurring of vision was mentioned by 266(57.7%) of women who heard about danger signs during pregnancy and this accounts for 37.6% of the seven hundred seven pregnant women included in the study. Out of a total number of women in the study 52.6, 37.6 and 23.5% of them mentioned vaginal bleeding, blurring of vision and swollen hands or face as a danger sign during pregnancy respectively. Retained placenta was the second most commonly mentioned key danger sign among women who heard about LAD danger signs 207(44.8%). Out of a total number of women in the study only 55.6, 29.3, 28.3, and 11.3% mentioned vaginal bleeding, retained placenta, prolonged labor, and convulsion as a danger sign during LAD respectively.

More than half of women who reported spontaneously that they heard about danger signs during the postpartum period 218(54.4%) mentioned foul-smelling vaginal discharge as a danger sign during the postpartum period. Out of a total number of women included in the study only 48.8, 30.8, 14.7% mentioned vaginal bleeding, foul-smelling vaginal discharge and high grade fever as a danger sign during the postpartum period respectively. Regarding the knowledge of the pregnant women during pregnancy, LAD, and the post-partum period more than a third of pregnant women 259(36.6%), a sizeable proportion of them 101(14.3%), and about a third of them 220 (31.1%) were knowledgeable on key danger signs during pregnancy, LAD and the postpartum period respectively (Table 4).

**Table 4:** Knowledge of pregnant women on key danger signs during pregnancy, LAD, and the post-partum period in Arba Minch Zuria Woreda, South Ethiopia, March 2015

Variables	Category	Frequency (%)
Ever heard of danger signs during pregnancy(N = 707)	Yes	461(65.2)
	No	246(34.8)
Know key pregnancy danger signs ⊕ (n = 461)	Vaginal bleeding	372(80.7)
	Swollen hands or face	168(36.4)
	Blurred vision	266(57.7)
	Others^a^	7(1.5)
Pregnancy danger signs can kill women(n = 461)	Yes	419(90.9)
	No	42(9.1)
Ever heard of LAD danger signs(N = 707)	Yes	462(65.3)
	No	245(34.7)
Know LAD danger signs(n = 462) ⊕	Severe vaginal bleeding	393(85.1)
	Prolonged labor	200(43.3)
	Convulsion	80(17.3)
	Retained placenta	207(44.8)
	Others^b^	15(3.3)
LAD danger signs can kill women(n = 462)	Yes	433(93.7)
	No	29(6.3)
Ever heard of postpartum danger signs(N = 707)	Yes	401(56.7)
	No	306(43.3)
Know postpartum danger signs(n = 401)	Severe vaginal bleeding	345(86.1)
	Foul-smelling vaginal discharge	218(54.4)
	High fever	104(25.9)
	Others^c^	7(1.7)
Postpartum danger signs can kill women(n = 401)	Yes	370(92.3)
	No	31(7.7)
Knowledgeable on pregnancy danger signs(N = 707)	Yes	259(36.6)
	No	448(63.4)
Knowledgeable on LAD danger signs(N = 707)	Yes	101(14.3)
	No	606(85.7)
Knowledgeable on postpartum danger signs(N = 707)	Yes	220(31.1)
	No	487(68.9)

^a^ excessive vomiting, cessation of fetal movement, etc

^b^ loss of consciousness, hand/foot presentation, etc

^c^ loss of consciousness, not able to breast feed, etc.

### Household food security status of the study participants

In this study, 323(45.7%) of pregnant women live in food secure households. Around a third of them 217 (30.7%) live in mild food insecure households, a sizeable proportion 97(13.8%) of them live in moderate food insecure households, and 70(9.9%) of them live in a severe food insecure households. Overall, 384 (54.3%) of the women live in food insecure households and the rest 323(45.7%) live in food secured households.

### Factors affecting birth preparedness and complication readiness among pregnant women in Arba Minch Zuria Woreda

The odds of being well prepared for birth and its complications was more than 2.2 times higher among women from high socioeconomic status than those from low socioeconomic status (AOR = 2.29(1.16, 4.54)).Similarly, it was more than 4.5 times higher among women with four and more antenatal visits than those who had less than four visits (AOR = 4.52(2.26, 9.02)). Women who received advice on preparation for birth and its complications during ANC were 1.84 times the odds of being well prepared for birth its complications than those who didn’t receive advice (AOR = 1.84(1.13, 3.01)). The odds of being well prepared for birth and its complications were 0.51 and 0.22 times lower among women with parity of two to four than those with a parity of one (AOR = 0.51(0.31,0.84) and AOR = 0.22(0.07,0.68)) respectively.

The FGD also revealed the importance of higher wealth/income where more than half of the discussants in all the groups (TBAs (60%), health professionals (75%), and husbands of pregnant women (55%) agreed that women who do not prepare for birth in time were those having less income and resources. The importance of complete attendance of ANC was also emphasized particularly by health professionals working in the area.

A 32 years old health officer working in the woreda said that *“In my decade experience in health profession here in this woreda and elsewhere, the major reason why my clients do not come early for birth is lack of resources …..even though we are providing a free maternity service here in the health center, some women could not afford to pay for their food and drink, though they finally come when the labor has already ended with complications; sometimes our staffs contribute from their pocket to send mothers to Arba Minch hospital when the ambulance we have gets occupied by another woman. People in the high lands here do not have access to markets, they do not produce cash crops, and are not having money, and hence could not save money”.*

Within the same discussion a 24 years midwife working in the same health center said*, “We often focus advice on birth preparations in later ANC visits but most women interrupt their ANC follow up”. Even though we were using female health development army leaders for peer education in the communities…women mock at us saying “you are working for your per diem”.*

Women who were knowledgeable on danger signs during LAD were 1.85 times the odds of being well prepared for birth and its complications than those who were not (AOR = 1.85(1.01, 3.44)). The odds of being well prepared for birth and its complications was about 0.26 times lower among women from food insecure households than those women from food secured households(AOR = 0.26(0.16, 0.42))(Table 5).

**Table 5:** Factors affecting birth preparedness and complication readiness in Arba Minch Zuria Woreda, South Ethiopia, March 2015

Variables	Category	BP/CR		OR 95%CI	AOR 95%CI
		Well prepared	Not well prepared		
Wealth status	High	89(12.6)	141(19.9)	1.74(1.16,2.62)^a^	2.29(1.16,4.54)^b^
	Medium-high	27(3.9)	96(13.6)	0.77(0.46,1.32)	2.19(0.93,5.18)
	Low-medium	42(5.9)	107(15.2)	1.08(0.67,1.74)	1.59(0.72,3.54)
	Low	54(7.6)	149(21.3)	1	1
Parity	One	78(13.4)	125(21.4)	1	1
	Two to Four	97(16.6)	235(40.3)	0.66(0.46,0.96)^a^	0.51(0.31,0.84)^b^
	Greater than or equal to 5	12(2.1)	36(6.2)	0.54(0.26,1.09)	0.22(0.07,0.68)^b^
Frequency of ANC visit	Less than four times	137(27.3)	295(58.7)	1	1
	Four and above visits	44(8.7)	26(5.2)	3.64(2.15,6.16)^a^	4.52(2.26,9.02) ^b^
Received advice on BP/CR	Yes	101(21.9)	148(32.1)	1.54(1.06,2.30)^a^	1.84(1.13,3.01)^b^
	No	65(14.1)	147(31.9)	1	1
Knowledge on LAD danger signs	Knowledgeable	44(6.2)	57(8.1)	2.02(1.31,3.09)^a^	1.85(1.01,3.44)^b^
	Not knowledgeable	168(23.8)	438(61.9)	1	1
Household food insecurity	Food insecure	79(11.2)	305(43.2)	0.54(0.33,0.87)^a^	0.26(0.16,0.42)^b^
	Food secured	133(18.8)	190(26.8)	1	1

*BP/CR* Birth Preparedness and Complication Readiness, *LAD* Labor and delivery, *COR* Crude odds ratio, *AOR* Adjusted odds ratio, *CI* Confidence interval

^a^ significant in bivariate analysis at p-value of less than 0.25

^b^ statistically significant association at p-value of less than 0.05 after adjusting for all the other variables in the model

In the FGDs too, the importance of inadequate food at home and its effect on birth preparation was emphasized by some respondents.

In the men FGD, a 35 years old man said *“we do not have adequate land, our children have already left to Addis Abeba and Arba Minch for weaving and shoe polishing, we need some more children because they serve us until they leave the area, we all strive to fulfill our belly, we expect a normal labor but sometimes we face bad days, but God will help us”.*

Regarding socio-cultural factors which hinder women from preparing for birth and its complications, the discussants in the FGDs reported that there was no strong culture and religious ground which prevents women from preparing for health facility delivery or giving birth in the health facilities. However, some of the participants raised issues related to exposure to sunlight, exposing their body to a male other than their husband and lack of the customary ceremonies at home immediately after birth like eating porridge, drinking coffee which they mentioned as minor as compared to women’s life, and they agreed that this days because of raising of awareness the community started to break this old traditions.

## Discussion

This community based study tried to assess prevalence of BP/CR and factors affecting it in randomly selected rural kebeles of Arba Minch Zuria Woreda, SNNPR. The prevalence of BP/CR was found to be 30%. The odds of being prepared for birth and its complications was higher among women from high economic class, frequency of antenatal care greater than or equal to four, who received advice on BP/CR,and who were knowledgeable on labor and delivery danger signs. However, it was lower for multiparous women and those from food insecure households.

In this study, with consideration of arrangements made on identification of the appropriate facility for delivery, mode of transportation, skilled attendant, saving money, only 30% of pregnant women were well prepared for birth and its complications. This was inadequate in comparison to the WHO standard that every pregnant woman should have a birth plan [4] implying increased efforts in the enhancement of BP/CR. However, it was relatively higher than a study conducted in Arsi Woreda, Central Ethiopia and Adigrat town, Northern Ethiopia, where only 16.5% and 22% of women in the studies were prepared for birth and its complications respectively [12, 14]. This could be related to the time of study where the Adigrat town study was conducted immediately after the implementation of focused antenatal care and this implies that women in the current study had ample of opportunities to hear about the important components of birth preparedness and complication readiness and would be benefited from the arrangements made by the government like free maternity service and ambulance service which was not in place by that time [16]. The difference with the study from Arsi Woreda of Oromiya region, Central Ethiopia [14] could be related to the sociocultural differences in the two regions and also the study subjects in the study were postnatal women who reported what they performed with possible loss of memory than the pregnant women in this study who report what they were doing by the time of the study.

Among the important components considered in the study, the most commonly made arrangement was saving money (54.2%) followed by identification of health facility for delivery (35.8%). This finding is consistent with findings from previous studies in India, Northern Nigeria, Uganda and Ethiopia [8–10, 12, 13]. This may be explained in relation to women’s knowledge that having money in hand enables them to buy the necessary materials, to have access for transportation at times of referrals in case of emergencies. However, it was higher than findings from recent studies conducted among pregnant women in Nigeria, Benin City, Edo state where only 29.4% [33, 34] of women saved money for emergency funds. In the Nigerian study the most common arrangement was made for identification of a health facility (98.3%) [34] and skilled provider for delivery (97.2%) [33]. This difference could be attributable to socio-cultural differences among the study participants.

The least arrangement was made for identification of blood donor (5%).It is consistent with findings from different studies across the world [8–10, 12, 13, 33, 34]. It could be related to the fact that the preparation particularly involves families and the community as a whole [5]. Bleeding during pregnancy, delivery and most importantly immediately after delivery is one of the major causes of maternal mortality across the world [7]. Women’s inadequate plan in relation to identifying potential blood donors can result in serious health consequences arising from severe vaginal bleeding during pregnancy, delivery and post-partum period. These unpredictable emergency events can occur in places were laboratory facilities may not be readily available to assess blood group and having this information can minimize time delays in accessing safe and appropriate blood for transfusion needed under such circumstances to save lives [33]. Lack of awareness on the important components of birth preparedness and its advantage was also supported by the FGD discussants (TBAs and husbands) as a barrier to women’s preparation. This implies that further efforts are required through provision of training to health care professionals and other community health workers (HEWs and HDA leaders).

The odds of being well prepared for birth and its complications was more than 2.2 times higher among women from high socioeconomic status than those from low socioeconomic status (AOR = 2.29(1.16, 4.54)). This implies that women from a higher socioeconomic situation could earn more money and hence are in a position to save money, arrange for transportation, and to have confidence in prioritization of health service matters as opposed to their counterparts.

Parity was the other predictor of birth preparedness and complication readiness. The odds of being well prepared for birth and its complications were 0.51 and 0.22 times lower among women with parity of two to four than those with a parity of one (AOR = 0.51(0.31,0.84) and AOR = 0.22(0.07,0.68)) respectively. This could be related to the less proportion of illiterate women among primiparas (23.1%) and high proportion (76.9%) of illiterate women in the multiparas as educational status is a strong predictor of BP / CR [11–14] even though it didn’t show a statistically significant association in this study. Besides this, the excitement from primiparas following pregnancy and the fact that they were less experienced could have influenced their better level of birth preparedness than others who might have gone through that road before.

In the study, the odds of being well prepared for birth and its complications was more than 4.5 times higher among women with four and more antenatal visits than those who had less than four visits (AOR = 4.52(2.26, 9.02)). This finding is consistent with findings from the Robe study from Oromiya region, Central Ethiopia [14]. Existing studies also showed that pregnant women who attended ANC were more likely to be prepared for birth and its complications than those who didn’t attend ANC [13–15, 33]. As the number of ANC visits increases, the likely that women get prepared for birth and its complications increases as there is advice on its components in each visit since birth preparedness and complication readiness is one of the interventions in the focused antenatal care. However, only 13.9% and 14.6% of women attended ANC four and more times and gave birth in the presence of a skilled professional in this study. In the FGDs of TBAs and husbands of women, lack of privacy and respect in health facilities was mentioned as one of the barriers to women’s commitment in preparing themselves for health facility delivery though maternity services are free of charge. This implies that health professionals should strive to keep ethical standards in service provision. Moreover, health care organizations has to strengthen their efforts in scaling up professional ethics as it is one of the guiding principles in the current health sector transformation plan [2].

Women who received advice on preparation for birth and its complications during ANC were 1.84 times the odds of being well prepared for birth its complications than those who didn’t receive advice (AOR = 1.84(1.13, 3.01)). This was in agreement with findings from the Adigrat town study. This could be explained by the effect of advice in raising the awareness of women on birth preparedness and complication readiness and which in turn resulted in a better practice of BP/CR [12].

Women’s knowledge of danger signs during each stage of childbearing (pregnancy, labor/birth, and postpartum) may help them recognize a life-threatening problem and take prompt action. It is the essential first step in the appropriate and timely referral to essential obstetric care [5].In this study, about a third of women had spontaneous knowledge of danger signs during pregnancy. This is relatively higher than the study conducted in Robe Woreda of Oromiya region, Central Ethiopia [14] where only 13% of women knew at least two key danger signs during pregnancy. A similar proportion of women in the study were knowledgeable on key danger signs during the postpartum period where as in the Robe study only 9.6%mentioned at least two key danger signs during postpartum period. This could be related to the difference in the study subjects where in the later study postnatal mothers were interviewed which implies that a possibility of memory loss will be there. However, the difference is negligible on knowledge of women during LAD where in this study only 14.3% were knowledgeable on key danger signs during LAD, where as in the Robe study 13% mentioned at least three key danger signs during LAD [14]. Women who were knowledgeable on danger signs during LAD were 1.85 times the odds of being well prepared for birth and its complications than those who were not (AOR = 1.85(1.01, 3.44)). This implies that women having knowledge on obstetric danger signs will have a fear that this danger signs could happen to them and get prepared for birth and its complications.

In this study, the odds of being well prepared for birth and its complications was about 0.26 times lower among women from food insecure households than those women from food secured households(AOR = 0.26(0.16, 0.42)). A recent study in Zimbabwe showed that household food insecurity was inversely associated with ANC service utilization and facility delivery among pregnant women [30], and the hypothesis that it could be operating through BP/CR in this study has come up with a strong independent association between the two(AOR = 0.26(0.16,0.42)). This may be explained by the fact that women in food insecure households strive for the fulfillments of their food condition than preparing themselves for birth and its complications because of the precedence of demand for food over demand for health care as food is the most basic need which cannot be postponed [29]. This implies improving food conditions at household level could enhance women’s preparation for birth and subsequently their facility delivery.

The study has few limitations to consider. The cross-sectional nature of this study may not explain temporal relationship between the outcome variable and certain explanatory variables. The state and months of pregnancy was self-reported by the woman or it was not confirmed by any diagnostic test. Similarly, pregnant women who were not registered in the family folders of the HEWs could underestimate the actual number of pregnant women living in the woreda. Thus, the findings of this study should be interpreted with consideration of these limitations.

## Conclusion

This study demonstrated that the prevalence of birth preparedness and complication readiness was inadequate in the study area. Socioeconomic/wealth status, parity, frequency of antenatal care, receiving advice on BPCR during ANC, knowledge on LAD danger signs, and household food insecurity were independent factors of birth preparedness and complication readiness. The main reasons given by the FGD discussants as a barrier to preparation for birth was lack of awareness, inadequate resources or income, and lack of privacy and respect in health facilities during delivery.

Thus, policy makers has to collaborate to enhance promotion of birth preparedness and complication readiness at different levels in the health sector by improving socio-economic and food security status of women. Community-based education on complete attendance of ANC should be strengthened on sustainable basis and antenatal care clinics should strengthen their advices on the components of BP/CR and obstetric danger signs with particular emphasis on multiparous pregnant women. The ministry of health in collaboration with regional health bureau, zonal health desks and other non-governmental partners has to strengthen its work on the provision of women friendly service in health institutions. Further studies assessing birth preparedness and complication readiness beyond individuals (pregnant or postnatal women) level at family, health facility and community level were recommended to bring further evidence in the efforts to end preventable maternal death.

## Additional file

Additional file 1: Raw data: It is an Excel data with the file name “revised additional file 1” which contains the raw data supporting the findings of this study. (XLS 1266 kb)

## Abbreviations

ANC: Ante -natal care
AOR: Adjusted odds ratio
BP/CR: Birth preparedness and complication readiness
EDHS: Ethiopian demographic and health survey
FANC: Focused antenatal care
FGD: Focused group discussion
FMOH: Federal Ministry of Health
HDA: Health development army
HSTP: Health sector transformation programme
LAD: Labor and delivery
MDG: Millennium development goal
MMR: Maternal mortality ratio
OR: Odds ratio
RMNCAH: Reproductive, maternal, neonatal, child, adolescent and youth health
SGDs: Sustainable development goals
SNNPR: Southern Nations and Nationalities and People’s Region
SPSS: Statistical package for social sciences
TBA: Traditional birth attendant
UN: United Nations
UNICEF: United Nations Children’s Fund
WHO: World Health Organization
